# Diode laser-activated sodium fluoride and galla chinensis extract as dentin biomodifiers: Effect on microhardness, resin tag length, and shear bond strength of universal adhesive to caries-affected dentin

**DOI:** 10.12669/pjms.42.9.16672

**Published:** 2026-09

**Authors:** Saeed Awod Bin Hassan, Saleh Ali Alqahtani, Nada Ahmad Alamoudi

**Affiliations:** 1 Saeed Awod Bin Hassan Associate Professor, Restorative Dental Sciences, College of Dentistry, King Khalid University, Abha, Kingdom of Saudi Arabia; 2 Saleh Ali Alqahtani Assistant Professor, Restorative Dental Science, College of Dentistry, King Khalid University, Abha, Kingdom of Saudi Arabia; 3 Nada Ahmad Alamoudi Assistant Professor, Restorative Dental Science, College of Dentistry, King Khalid University, Abha, Kingdom of Saudi Arabia

**Keywords:** Sodium fluoride, Galla chinensis extract, Bioactive glass nanoparticles, Caries-affected dentin

## Abstract

**Objective:**

To evaluate the effects of dentin biomodifiers sodium fluoride (NaF), blue diode laser (DL) irradiated NaF, Galla chinensis extract (GCE), and bioactive glass nanoparticles (BAGNPs), on the microhardness (MH), shear bond strength (SBS), and resin tag length (RTL) of a self-etch (SE) universal adhesive system bonded to caries-affected dentin (CAD).

**Methodology:**

Eighty disinfected permanent molars were included in the study. The present in vitro study was approved by the Ethical Committee of King Khalid University IRB-KKU-COD-ETH-0531. The study duration was four months, from 15^th^ September 2025 to 15^th^ January 2026. CAD was confirmed through visual, tactile, and staining methods. Samples were randomly divided into five groups based on the type of surface modifier(n=16). Group-1: No biomodification, Group-2: NaF, Group-3: Blue DL irradiated NaF, Group-4: BAGNPs, and Group-5: GCE. Surface MH testing was conducted using a Vickers hardness tester (n=4). RTL was identified using a SEM (n=2). The universal adhesive was applied using the SE technique, followed by composite buildup (n=10). SBS testing was performed using a Universal Testing Machine. The fractured specimens were evaluated under a stereomicroscope. ANOVA and Tukey’s post hoc test were used to compare the mean MH, RTL, and bond strength values (p<0.05).

**Results:**

Group-3 (DL-irradiated NaF) exhibited the highest MH, whereas the lowest MH was observed in Group-1. Group-5 (GCE) demonstrated the maximum RTL (67.02±3.76μm) and the highest SBS (10.27±0.44 MPa).

**Conclusion:**

Galla chinensis extract and diode laser-irradiated sodium fluoride, when used independently, each enhanced the SBS and RTL of the self-etch universal adhesive to caries-affected dentin. Only blue laser-irradiated sodium fluoride was effective in augmenting the MH of caries-affected dentin.

## INTRODUCTION

In restorative dentistry, minimally invasive dentistry (MID) has gained attention as a caries-management strategy. This approach advocates selective removal of infected dentin, eliminating heavily contaminated tissue while preserving caries-affected dentin (CAD).^1^ Notably, CAD retains remineralization potential due to its intact collagen matrix, reduced bacterial load, and distinct morphology.^2^ Compared to sound dentin, bonding to CAD forms a more complex hybrid layer, with a wider zone of exposed collagen fibrils and diminished shear bond strength (SBS).^3^ Thus, pre-treatment of CAD with dentin bio-modifiers before restorative procedures is advocated to enhance substrate quality and bonding performance.

Fluoride plays a critical role due to its anticariogenic properties, preventing caries by inhibiting demineralization and promoting remineralization. The effect of fluoride pretreatment on adhesive bonding is debated. Evidence suggests fluoride ions from NaF may preserve the hybrid layer by protecting collagen fibrils from enzymatic degradation, explaining enhanced bond strength.^4^,^5^ Laser technology has emerged as a promising adjunct in restorative dentistry. Research by Liu et al. showed that a diode laser (DL), used alone or with topical fluoride, offers a straightforward, minimally invasive method for caries prevention by reducing solubility and demineralization susceptibility of dental hard tissues.^5^ Fahim et al. found blue laser irradiation of enamel increases fluoride retention by 42.3% after 7 days, compared to 12.3% in non-laser-irradiated enamel.^6^ Despite these outcomes, the potential of blue DL irradiated NaF for CAD biomodification and its impact on resin tag length (RTL) bond strength and MH remains unclear, highlighting the need for further well-designed investigations.

Recent advancements in bioactive and multifunctional materials show significant potential in managing dental caries. Bioactive glass nanoparticles (BAGNPs) are known for forming hydroxyl carbonate apatite (HCA) in simulated body fluids and are studied for tissue engineering, bone healing, and dentin remineralization.^7^ Incorporating BAGNPs into dental adhesives may enhance bonding by aiding remineralization and providing antibacterial properties.^8^ However, the effects of BAGNPs as dentin biomodifiers on dentin hardness, RTL, and bond strength with universal adhesives are not well understood and need more research.

Natural products, known for their diverse chemical composition, biological origins, potent pharmacological properties, and low toxicity, have been a crucial source of therapeutic agents for over three millennia.^9^ Among these, Galla chinensis extract (GCE), a traditional herbal remedy in Chinese medicine, has gained attention in dentistry for its anticariogenic and antibacterial potential.^10^ GCE shows significant collagen crosslinking potential, primarily due to its hydrolyzable tannins, especially gallotannins like gallic acid and methyl gallate. It also contains trace inorganic elements like calcium (Ca), phosphorus (P), iron (Fe), manganese (Mn), and zinc (Zn), which may aid remineralization.^11^ However, despite these promising properties, evidence on its effect on the MH, RTL, and bond integrity of the 8th-generation self-etch universal adhesive to CAD is limited, requiring further exploration.

The current study is predicated on the hypothesis that there will be no significant difference in the MH of CAD pretreated with sodium fluoride (NaF), blue DL irradiated NaF, GCE, and BAGNPs compared to the control. Additionally, it is anticipated that the results for RTL and SBS of CAD biomodified using various agents will be comparable to those of the control. Consequently, the study aimed to evaluate the impact of different dentin biomodifiers on the MH, RTL, and bond strength of universal adhesive to CAD.

## METHODOLOGY

A total of eighty extracted permanent molars were disinfected using a 0.1% thymol solution (DR Thym™, InnuScience, Canada). After disinfection, the specimens were thoroughly rinsed under running water, and surface deposits were removed with an ultrasonic scaler (Woodpecker® UDS-K LED, China). The teeth selected for the study met the ICDAS criteria scores of four and five. CAD was identified through a combination of visual examination, tactile evaluation with a dental explorer, and staining with a 0.5% basic fuchsin caries detector dye (Merck, Germany). All teeth were examined under a stereomicroscope at 40 × magnification to ensure suitability. Specimens with enamel defects, cracks, fractures, discoloration, or developmental anomalies were excluded. The present in-vitro study was approved by the Ethical Committee of King Khalid University IRB-KKU-COD-ETH-0531. The study duration was four months, from 15^th^ September 2025 to 15^th^ January 2026. Each tooth was embedded in acrylic resin (Duracryl PLUS, Spofa Dental, Czechia) up to the cementoenamel junction. The occlusal enamel was sectioned perpendicular to the tooth’s long axis using a high-speed fine diamond bur mounted on a high handpiece (NSK EX-6B, NSK, Japan) under continuous air–water irrigation, exposing the dentin surface and creating a uniform smear layer.

### Preparation of GCE-containing cement:

*Galla chinensis* (origin: Sichuan Province, China) was procured from China Tong Ren Tang Drugstore (Chengdu, China). Approximately 1 kg of the material was oven-dried at 60°C for 72 hours, followed by pulverization into a fine powder. The powdered sample was then subjected to aqueous extraction by adding 600 mL of distilled water and maintaining continuous stirring at 65 °C for 10 hours. The resulting mixture was filtered, and the residue was re-extracted under identical conditions to ensure maximum yield. The combined filtrates were subsequently treated with 500 mL of 95% ethanol, followed by filtration. The solvent was removed by evaporation, and the concentrated extract was finally lyophilized to obtain a dry powdered form. To prepare a 10% (w/v) solution, 10 g of GCE powder was dissolved in 100 mL of distilled water. The solution was stirred for 20–30 minutes at room temperature to ensure complete dissolution and then filtered through Whatman No.1 filter paper to remove any undissolved particles. The prepared solution was stored in an amber bottle at 4°C until further use.

### Grouping of the teeth:

Specimens were randomly divided into five groups according to the type of surface modifier used (n=16)

### Group-1: No biomodification:

In this group, no biomodification was performed.

### Group-2: 5% NaF:

The CAD surface was pre-treated with 5% sodium fluoride varnish (Charm Varnish^®^ – Dentkist, Korea) with the help of an applicator tip and left undisturbed for 5 minutes.^12^

### Group-3: Blue DL irradiated NaF:

NaF was applied in a manner identical to that described for Group-2. Subsequently, the treated dentin surface was irradiated using a blue diode laser (Sirona, USA) operating at a wavelength of 445 nm, with an output power of 0.3 W for 60 seconds in continuous-wave mode. The laser was delivered through a 320 μm fiber optic tip, corresponding to a spot size of 0.196 cm² and an energy density of 91.8 J/cm². Irradiation was performed at a standardized distance of 1 mm from the tooth surface to ensure uniform energy distribution.^13^ Intrapulpal temperature was continuously monitored during irradiation, positioned at the pulpal wall, and the maximum recorded temperature rise did not exceed the 5.5°C threshold

### Group-4: 2.5 wt% BAGNPs:

BAGNPs were dispersed in ethanol at a concentration of 2.5% (v/v) and subjected to ultrasonication for 3 minutes in an ice bath using an ultrasonic probe (Sonoplus UW2200; Bandelin Electronic GmbH, Berlin, Germany) to ensure uniform dispersion. The stability of the nanoparticle suspension was subsequently assessed using a separation analysis device (LUMiReader® 416.1; LUM GmbH, Berlin, Germany). The CAD surface was then treated with the prepared BAGNPs suspension for 10 seconds, followed by gentle air-drying.^14^

### Group-5: 10% GCE:

The prepared 10% GCE was applied on the surface using microbrush and on the surface for one minute.^10^

### Surface microhardness testing:

The Vickers hardness of the dentin surface was measured using a digital tester. This device featured a square-based pyramidal diamond indenter for precise substrate penetration. Three indentations, each 1 mm apart, were noted. A 100-g load was applied perpendicularly for 15 seconds at each site. For each specimen, three readings were averaged to report the Vickers Hardness Number (VHN, g/mm²).

### Bonding of adhesive and composite:

Single-dose capsules of Futurabond DC (VOCO, Germany) were activated and mixed per the manufacturer’s protocol. The adhesive was applied to dentin with a microbrush and rubbed for 20 seconds to enhance tubule penetration. After gentle air-drying for five seconds, the adhesive layer was light-cured for 10 seconds using an LED unit (Demi Ultra™, Kerr Corporation, USA). Samples were subsequently restored with a 2×3mm light-curing composite (Beautifil, Shofu) in a Teflon mold and cured for 10 seconds with an LED unit.

### Storage and Aging Protocol:

Restored specimens were initially incubated in distilled water at 37 °C for 24 hours to ensure complete curing of both the adhesive and composite. Thereafter, they underwent thermocycling for 10,000 cycles between 5 °C and 55 °C, maintaining a dwell time of 30 seconds in each bath. The transfer time between the hot and cold baths was standardized at five seconds.

### RTL assessment using SEM:

Two samples per group were randomly selected to measure RTL. Each tooth was sectioned longitudinally. The samples were treated with 37% phosphoric acid for 15 seconds and rinsed with water for 30 seconds. The resin–dentin interface was examined using SEM analysis through both qualitative and quantitative methods, namely visual inspection and RTL measurement. The lengths of resin tags were measured directly from SEM images using a ruler standardized with the image scale bar. Qualitative assessment was conducted using a scoring system based on the four-step (0–3) classification proposed by Ferrari and colleagues.^15^

### SBS testing:

Each specimen was individually mounted in semi-open front hydraulic wedge grips of an Instron Universal Testing Machine (MTS, Eden Prairie, USA) and securely stabilized before testing. Shear load was applied at the composite–CAD interface using a knife-edged blade with a thickness of 0.5 mm. The crosshead speed was set at 1 mm/min, and loading continued until specimen failure.

### Fracture pattern analysis:

The fractured specimens were evaluated under a stereomicroscope (Optika SZM-LED2 Stereomicroscope, Optika, Italy) at 40× magnification to determine the mode of failure. The failure patterns were classified as adhesive, cohesive, and admixed.

### Statistical analysis:

Mean and standard deviation were calculated for each experimental group. One-way analysis of variance was performed to compare the mean MH, RTL, and bond strength values among the groups. Significant findings were further analyzed with Tukey’s post hoc test, p < 0.05.

## RESULTS

### Microhardness of CAD:

The mean MH values of CAD after pretreatment with different dentin biomodifiers are presented in Table-I. Among all the groups, Group-3 (DL-irradiated NaF) demonstrated the maximum MH (40.43 ± 2.54). In contrast, the minimum MH was observed in Group-1 (no biomodification) (19.22***±***3.45). Intergroup comparison revealed that DL-irradiated NaF showed significantly higher MH than all other groups (p < 0.05). However, no statistically significant difference was observed among the control, Group-2 (NaF) (19.15 ± 4.11), Group-4 (BAGNPs) (19.01 ± 3.12), and Group-5 (GCE) (19.11 ± 2.21) groups. (p > 0.05)

**Table-I:** MH, RTL, and SBS of self-etch universal adhesive to CAD after using different dentin biomodifiers.

Dentin Biomodifier	Microhardness (MH)	Resin Tag Length (RTL)		Shear Bond Strength (SBS)	p-value!
	Mean ± SD (VHN)	Visual Score	Mean ± SD (μm)	Mean ± SD (MPa)	
Group-1: No biomodification	19.22 ± 3.45 ^B^	1	48.12 ± 3.12 ^A^	8.33 ± 0.42 ^A^	p<0.05
Group-2: NaF	19.15 ± 4.11 ^B^	1	49.01 ± 2.56 ^A^	8.41 ± 0.36 ^A^	
Group-3: Blue DL irradiated NaF	40.43 ± 2.54 ^A^	3	65.12 ± 4.55 ^B^	10.21 ± 0.54 ^B^	
Group-4: BAGNPs	19.01 ± 3.12 ^B^	1	50.01 ± 3.02 ^A^	8.36 ± 0.32 ^A^	
Group-5: GCE	19.11 ± 2.21 ^B^	3	67.02 ± 3.76 ^B^	10.27 ± 0.44 ^B^	

!ANOVA; NaF: Sodium fluoride; DL: Blue diode laser; GCE: Galla chinensis extract;

BAGNPs: Bioactive glass nanoparticles; VHN: Vickers hardness number

Distinct superscripts (A, B) denote significant intergroup differences (p<0.05).

### RTL assessment:

The mean RTL of adhesive to biomodified CAD is presented in Table-I. Among all the tested groups, Group-5 (GCE) exhibited the maximum RTL (67.02 ± 3.76 μm). In contrast, the minimum RTL was observed in Group-1 (no biomodification) (48.12 ± 3.12 μm). Group-1, Group-2 (NaF) (49.01 ± 2.56 μm), and Group-4 (BAGNPs) (50.01 ± 3.02 μm) presented comparable outcomes of RTL with score 1 on visual examination(p > 0.05). Likewise, Group-5 (GCE) (67.02 ± 3.76 μm) and Group-3 (DL-irradiated NaF) also resulted in comparable and longer RTL with score 3 on visual examination when compared to other tested biomodified groups (p > 0.05) Fig.1.

**Fig.1:**
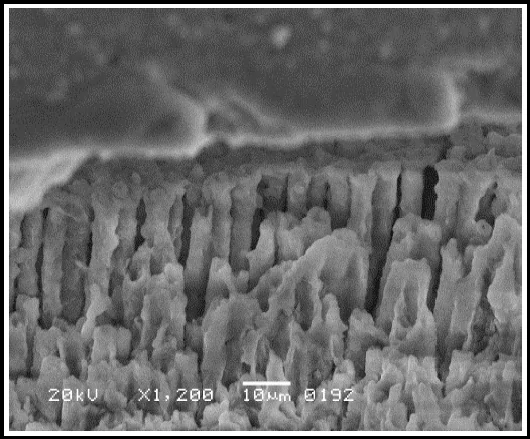
SEM Image of CAD biomodified using GCE.

### SBS analysis:

The mean SBS of the SE universal adhesive bonded to biomodified CAD is presented in Table-I. Among all the tested groups, Group-5 (GCE) demonstrated the maximum bond strength (10.32 ± 0.44 MPa). In contrast, the minimum bond strength was observed in Group-1 (No biomodification) (8.33±0.42 MPa). Group-5 (GCE) (10.27 ± 0.44 MPa) and Group-3 (DL-irradiated NaF) (10.21 ± 0.54 MPa) showed comparable and significantly higher bond strength to other CAD modified groups (p > 0.05). Likewise, control, Group-2 (NaF) (8.41± 0.36 MPa), and BAGNPs (8.36 ± 0.32MPa) also displayed comparable outcomes of SBS (p > 0.05).

### Fracture mode analysis:

Fracture pattern results are displayed in Fig.2. Blue-light irradiated NaF and GCE pretreated CAD samples predominantly demonstrated cohesive failures, whereas the remaining experimental groups mainly exhibited mixed (admixed) failure patterns.

**Fig.2:**
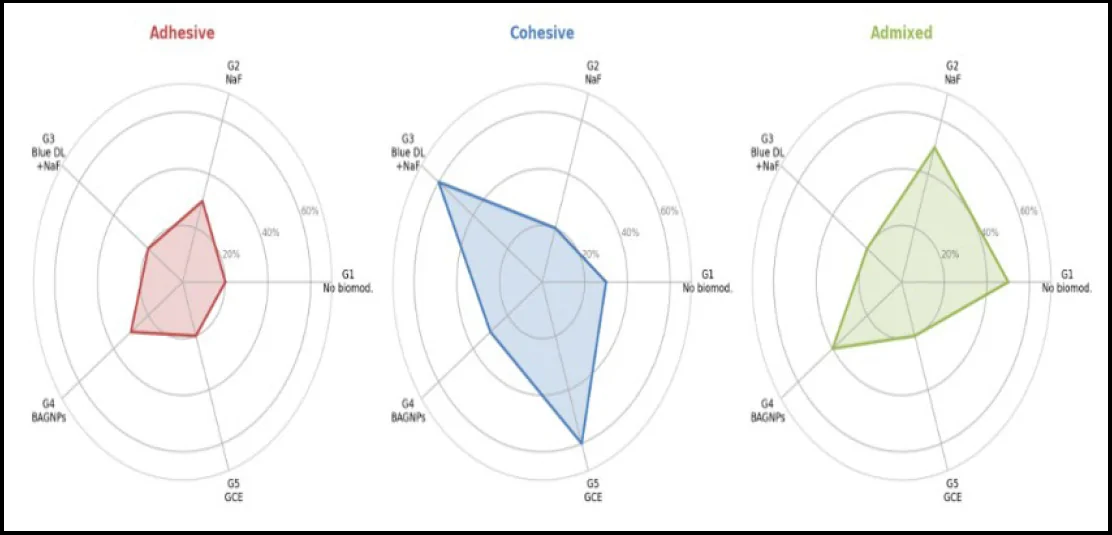
Fracture pattern distribution- Radar charts by fracture modes.

## DISCUSSION

The present study was predicted on two null hypotheses: (1) that pretreatment of CAD with NaF, blue DL-irradiated NaF, GCE, and BAGNPs would produce no significant difference in MH compared to the untreated control; and (2) that the RTL and SBS of a self-etch universal adhesive to biomodified CAD would remain comparable across all experimental groups relative to the control. The findings of the present study, however, necessitated the partial rejection of both null hypotheses. Pretreatment with DL-irradiated NaF resulted in a statistically significant increase in MH compared to the control group, thereby refuting the first null hypothesis. Furthermore, both the GCE and DL-irradiated NaF groups demonstrated significantly greater RTL and SBS values relative to the control, leading to the rejection of the second null hypothesis for these specific treatment modalities.

The current analysis revealed that GCE-modified dentin resulted in significantly longer RTL and the highest SBS, although it did not enhance dentin MH. This finding can be elucidated by existing literature, which suggests that the hydroxyl and carboxyl groups in GCE form multiple hydrogen bonds with acidic and basic amino acid side chains in collagen.^11^ This interaction mitigates biodegradation by host endogenous enzymes, such as matrix metalloproteinases (MMPs) and collagenase.^6^ Furthermore, it inhibits the dissolution of Ca^2+^ in the dentin, thereby stabilizing collagen fibers. Stabilized collagen fibrils prevent the collapse of the demineralized dentin network, maintaining open interfibrillar spaces.^9^ This facilitates deeper penetration of adhesive monomers into dentinal tubules, resulting in longer resin tags and the highest SBS.

NaF biomodified CAD samples exhibited MH, RTL, and SBS comparable to the control group. This finding is consistent with earlier laboratory research by Biria and colleagues, which indicated that applying 5% NaF varnish does not typically result in an immediate increase in dentin MH. However, a significant enhancement is observed over a longer period, such as around 45 days, suggesting a gradual remineralization effect. Furthermore, delaying composite resin restoration for 10 days after applying 2% NaF gel has been reported to improve bond strength beyond that of the control group.^16^ In contrast, the NaF group treated with a blue diode laser demonstrated the highest MH, RTL, and SBS. This observation aligns with in vitro analysis by Hashemikamangar and colleagues.^13^ They reported that when NaF is applied to dentin and then exposed to blue light (≈470 nm), several changes can occur. The light energy may enhance the diffusion of fluoride ions, promoting the formation of fluorapatite by replacing carbonate or hydroxyl groups with fluoride, ultimately resulting in the highest MH. Fluoride also stabilizes the hybrid layer, reduces collagen degradation, enhances SBS, and extends the resin tags.^13^

### Strength:

When used as a dentin biomodifier, BAGNPs yielded MH, RTL, and bond strength values similar to the control group. This aligns with a study by Saeed Mosavinasab et al., showing that a 20% BAGNPs suspension caused only minor, statistically insignificant SBS improvement. Previous research indicates that BAGNPs suspensions are more effective with etch-and-rinse adhesives than SE adhesives.^17^ Studies show BAGNPs suspensions enhance bond strength, MH, and hybrid layer elasticity. The mechanism involves forming silanol (Si–OH) groups on the BAG surface, regulating ionic exchange and pH, creating an alkaline environment that promotes apatite nucleation and growth.^18^ Variations in study results may stem from differences in experimental design, BAG particle size, concentration, and composition, and dentin rehydration methods post-BAG application.

### Limitations:

This laboratory-based investigation has limitations to consider when interpreting findings. The complex biological, chemical, and mechanical conditions of the oral environment cannot be fully replicated in vitro, possibly limiting clinical applicability. Additionally, factors like particle size, concentration, and compositional variations of BAGNPs can significantly influence bonding outcomes. The brief application duration in this study may have been insufficient for optimal reinforcement of the demineralized dentin matrix.

### Novelty and Contribution to Dental Literature:

Applying GCE, a natural polyphenol-rich plant extract known for collagen crosslinking and MMP inhibitory properties, to CAD as a bonding pretreatment remains largely unexplored. Integrating blue diode laser irradiation with sodium fluoride as a synergistic strategy for dentin biomodification is relatively novel. The rationale—that laser energy enhances fluoride ion penetration and hydroxyapatite fluoridation, increasing dentin hardness and potentially improving bonding—is mechanistically plausible but not extensively documented. Most research on collagen crosslinking and biomodification in caries-affected dentin has used etch-and-rinse (ER) adhesives, as phosphoric acid etching aligns mechanistically with crosslinking agents requiring exposed collagen. Evaluating biomodifiers with a self-etch universal adhesive is clinically relevant, given the increasing preference for self-etch systems due to their simplicity and reduced technique sensitivity. The study’s most significant contribution is finding that the microhardness of caries-affected dentin does not directly correlate with SBS outcomes, challenging a widely held assumption in restorative dentistry.

## CONCLUSION

Galla chinensis extract and diode laser-treated sodium fluoride, applied as separate biomodifiers, were each shown to improve the shear bond strength and resin tag length of self-etch universal adhesive when applied to dentin affected by caries. However, only sodium fluoride exposed to blue laser irradiation enhances the microhardness of carious dentin.

### Authors’ Contribution:

**SABH:** Data collection, study design, and Conceptualization.

**SAA, NAA, and SABH:** Manuscript writing, data analysis, and final manuscript approval.

All authors are responsible and accountable for the accuracy and integrity of the present work.
